# Supplementary material for: High-quality histochemistry, immunohistochemistry, and immunofluorescence on xylene- and formalin-free paraffin-embedded tissues
Source: PLoS One. 2026 Feb 5;21(2):e0340480. doi: 10.1371/journal.pone.0340480 (PMC12875444; doi:10.1371/journal.pone.0340480)

NFPE skin P63		NFPE skin P63		FFPE skin P63			NFPE skin ker 5	
Min#	Value	Min#	Value	Min#	Value		Min#	Value
66	189	62	193	62	193		92	163
62	193	64	191	65	190		90	165
67	188	61	194	63	192		87	168
62	193	69	186	68	187		87	168
64	191	62	193	65	190		84	171
67	188	60	195	62	193		94	161
59	196	63	192	64	191		92	163
61	194	65	190	65	190		97	158
69	186	72	183	63	192		87	168
61	194	72	183	62	193		83	172

scale 0-255 > darkest is 0 > 255-measurement = Staining intensity

		P63	P63	P63		Ker5	
		NFPE	XFPE	FFPE		NFPE	
	avg	191,2	190	191		165,7	
	st dev	3,3	4,4	1,9		4,5	
T-Test (paired;2;2)							
P63	NFPE	x	x	x		2,23E-11	****p
P63	XFPE	0,5016	x	x		3,96E-10	****p
P63	FFPE	0,9347	0,482	x		2,56E-12	****p

Significance levels T-test
n.s. p > 0.05 The difference is not significai n.s.
*p < 0.05 (one star): The difference is significant. Chance of coincidence lower than 5%.
**p < 0.01 (two stars): The difference is highly significant. Chance of coincidence lower than 1%.
***p < 0.001 (three stars): The difference is highly significant. Chance of coincidence lower than 0.1%.
****p < 0.0001 (four stars): The difference is extremely significant. Chance of coincidence lower than 0.01%.

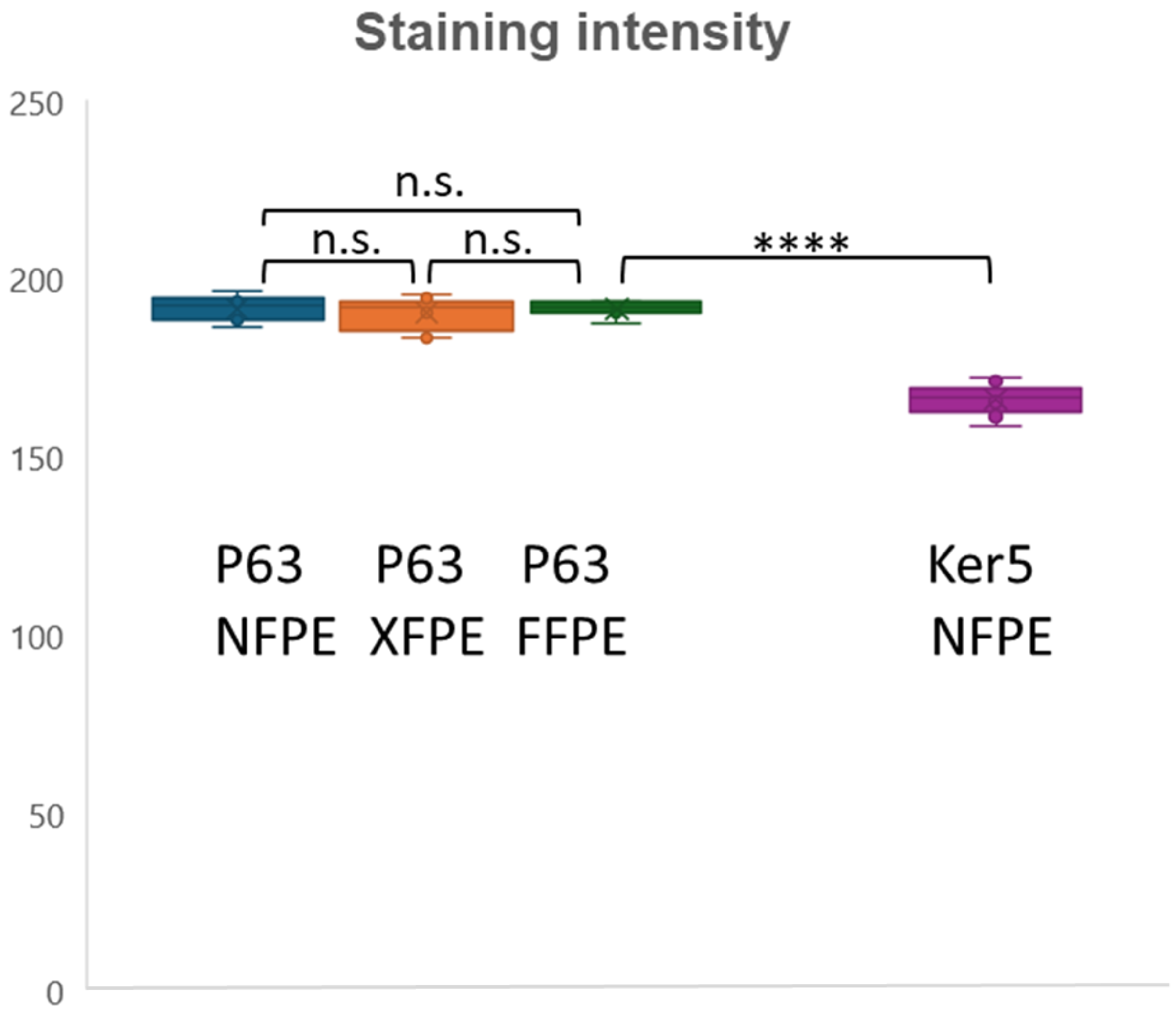

Supplement: S5 Fig — (PDF) [file pone.0340480.s005.pdf]
