# Supplementary material for: High-quality histochemistry, immunohistochemistry, and immunofluorescence on xylene- and formalin-free paraffin-embedded tissues
Source: PLoS One. 2026 Feb 5;21(2):e0340480. doi: 10.1371/journal.pone.0340480 (PMC12875444; doi:10.1371/journal.pone.0340480)
Supplement: Attachment — Submitted filename: Response to reviewers_Niemantsverdriet et al.docx [file pone.0340480.s006.docx]

PONE-D-25-46295

High-quality histochemistry, immunohistochemistry, and immunofluorescence on xylene- and formalin-free paraffin-embedded tissues

PLOS ONE

Dear Dr. Hoogland,

Thank you for submitting your manuscript to PLOS ONE. After careful consideration, we feel that it has merit but does not fully meet PLOS ONE’s publication criteria as it currently stands. Therefore, we invite you to submit a revised version of the manuscript that addresses the points raised during the review process.

In your revised manuscript, please ensure that you address the following concerns raised by Reviewers 1 and 2. While both reviewers agree that your study presents an innovative, well-written, and highly relevant advance in formalin- and xylene-free tissue processing, several revisions are necessary to strengthen methodological rigor, reproducibility, and quantitative support for your conclusions. While you should address all reviewer comments completely, I will point out the following critical comments:

Specify the number of biological and technical replicates analyzed for each staining approach.

A: The number of biological replicates is the number of different tissues used as specified in tables 4, 5 and 6. For each tissue, we performed 2 technical replicates after optimization. We now also explain this in the methods section of the revised manuscript, and mention this in the caption of the tables.

Identify who performed morphological assessments (e.g., pathologists, trained reviewers) and state whether evaluations were blinded.

A: The assessments of all stainings were initially performed blinded by an expert reviewer (specialized technician), followed by a not-blinded assessment by a general pathologist and finally verified by a specialized pathologist. We now explain this in the methods section of the revised manuscript and mention this in the caption of tables 4, 5, and 6.

Include counts or percentages of marker-positive cells (e.g., P63⁺), signal intensity measures, or reproducibility scoring.

A: We quantified P63 signal intensity scoring in FFPE, XFPE, and NFPE stainings as requested and show in a new supplementary figure (S4) that we were able to obtain P63 staining intensities in NFPE, XFPE and FFPE tissues that show no statistical difference.

Include appropriate statistical analyses to support statements of equivalence or similarity between FFPE, NFPE, and XFPE groups.

A: As mentioned above, we quantified P63 signal intensity scoring between FFPE, NFPE, and XFPE groups and showed that we were able to achieve signal intensities that show no statistical difference. Observations in this manuscript were initially performed double-blinded by an expert reviewer (specialized technician) and verified (not-blinded) by both a general pathologist and a specialized pathologist. We achieved a 100% inter-observer agreement and we now describe that in the revised manuscript and mention this in the caption of tables 4, 5, and 6. We now more clearly describe in the revised manuscript why we used observational analysis as preferred method for assessment of stainings. We added a new reference (Elliot et. all.) describing that because tissues lack homogeneity, already in unprocessed form, the resulting stainings also lack homogeneity, this is independent of tissue processing method. Therefore, for a general, novel study, testing a large quantity of histochemical, immunohistochemical, and immunofluorescence stainings, thorough observational analysis is more appropriate than statistical comparison.

Discuss the diagnostic implications of markers that failed in NFPE (CD68, PAX-8, TDT) and note possible solutions such as alternative antibody clones.

A: In the revised manuscript, we discuss diagnostic implications of the markers that failed and note possible solutions.

Provide commentary on the altered collagen morphology observed in NFPE skin samples, and if possible, evaluate whether picrosirius red with polarized light can distinguish collagen subtypes in NFPE-processed tissues.

A: In our laboratory and in the other diagnostic pathology laboratories in the Netherlands, the picrosirius red staining is not used for diagnostics. To assess collagen morphology in diagnostics, we use Azan, Masson Goldner (trichrome) and Elastica van Gieson (EvG) stainings. We performed these stainings on other tissue types in our original manuscript, but not yet on skin NFPE. In the revised manuscript, we performed these stainings on skin NFPE samples and added a new figure (Figure 2 in the revised manuscript). We describe the findings in a new paragraph. We did not observe altered (collagen) morphology.

Improve your methods section to provide sufficient methodological detail to allow replication in other laboratories.

A: We have now extended the methods section in the revised manuscript and explained the NFPE process in detail.

Please submit your revised manuscript by Nov 30 2025 11:59PM. If you will need more time than this to complete your revisions, please reply to this message or contact the journal office at [plosone@plos.org](mailto:plosone@plos.org). When you're ready to submit your revision, log on to <https://www.editorialmanager.com/pone/> and select the 'Submissions Needing Revision' folder to locate your manuscript file.

Please include the following items when submitting your revised manuscript:

- A rebuttal letter that responds to each point raised by the academic editor and reviewer(s). You should upload this letter as a separate file labeled 'Response to Reviewers'.
- A marked-up copy of your manuscript that highlights changes made to the original version. You should upload this as a separate file labeled 'Revised Manuscript with Track Changes'.
- An unmarked version of your revised paper without tracked changes. You should upload this as a separate file labeled 'Manuscript'.

If you would like to make changes to your financial disclosure, please include your updated statement in your cover letter. Guidelines for resubmitting your figure files are available below the reviewer comments at the end of this letter.

If applicable, we recommend that you deposit your laboratory protocols in protocols.io to enhance the reproducibility of your results. Protocols.io assigns your protocol its own identifier (DOI) so that it can be cited independently in the future. For instructions see: [https://journals.plos.org/plosone/s/submission-guidelines#loc-laboratory-protocols](https://track.editorialmanager.com/CL0/https:%2F%2Fjournals.plos.org%2Fplosone%2Fs%2Fsubmission-guidelines%23loc-laboratory-protocols/1/010f0199f0164187-b2779843-ba5e-48b7-a0db-e390769d2d97-000000/p3obihwJeX-jB4xFtjN_6bpTm2AxbjCENjS9xiFQJ6M=233). Additionally, PLOS ONE offers an option for publishing peer-reviewed Lab Protocol articles, which describe protocols hosted on protocols.io. Read more information on sharing protocols at [https://plos.org/protocols?utm_medium=editorial-email&utm_source=authorletters&utm_campaign=protocols](https://track.editorialmanager.com/CL0/https://plos.org/protocols).

We look forward to receiving your revised manuscript.

Kind regards,

Jordan Robin Yaron, Ph.D.
Academic Editor
PLOS ONE

Journal Requirements:

When submitting your revision, we need you to address these additional requirements.

1. Please ensure that your manuscript meets PLOS ONE's style requirements, including those for file naming. The PLOS ONE style templates can be found at

<https://journals.plos.org/plosone/s/file?id=wjVg/PLOSOne_formatting_sample_main_body.pdf> and

[https://journals.plos.org/plosone/s/file?id=ba62/PLOSOne_formatting_sample_title_authors_affiliations.pdf](https://track.editorialmanager.com/CL0/https://journals.plos.org/plosone/s/file)

2. Thank you for stating the following in the Competing Interests section:
I have read the journal's policy and the authors of this manuscript have the following competing interests:

Agnes Marije Hoogland is included as one of the inventors in the Tispa patent WO 2024/117901 A1 of Tispa Medical B.V. and Stichting Isala Klinieken on the Non-Fixed Paraffin-Embedded (NFPE) technique, with additional patents pending. The other authors do not report a conflict of interest.

Please confirm that this does not alter your adherence to all PLOS ONE policies on sharing data and materials, by including the following statement: "This does not alter our adherence to PLOS ONE policies on sharing data and materials.” (as detailed online in our guide for authors <http://journals.plos.org/plosone/s/competing-interests>). If there are restrictions on sharing of data and/or materials, please state these. Please note that we cannot proceed with consideration of your article until this information has been declared.

Please include your updated Competing Interests statement in your cover letter; we will change the online submission form on your behalf.

A: We have now included the statement in the cover letter and in the revised manuscript.

3. Please amend the manuscript submission data (via Edit Submission) to include author Marije Hoogland

A: We added Agnes Marije Hoogland to the manuscript submission data

4. Please amend your authorship list in your manuscript file to include author Agnes Marije Hoogland

A: We have now added Agnes to Marije Hoogland’s name in the revised manuscript.

5. Please include captions for your Supporting Information files at the end of your manuscript, and update any in-text citations to match accordingly. Please see our Supporting Information guidelines for more information: <http://journals.plos.org/plosone/s/supporting-information>.

A: We have now added captions for supporting information files in the revised manuscript.

6. If the reviewer comments include a recommendation to cite specific previously published works, please review and evaluate these publications to determine whether they are relevant and should be cited. There is no requirement to cite these works unless the editor has indicated otherwise.

A: There was no specific recommendation to cite previously published work

[Note: HTML markup is below. Please do not edit.]

Reviewers' comments:

Reviewer's Responses to Questions

**Comments to the Author**

1. Is the manuscript technically sound, and do the data support the conclusions?

The manuscript must describe a technically sound piece of scientific research with data that supports the conclusions. Experiments must have been conducted rigorously, with appropriate controls, replication, and sample sizes. The conclusions must be drawn appropriately based on the data presented.

Reviewer #1: Yes

Reviewer #2: Partly

2. Has the statistical analysis been performed appropriately and rigorously?

Reviewer #1: Yes

Reviewer #2: No

3. Have the authors made all data underlying the findings in their manuscript fully available?

The [PLOS Data policy](http://track.editorialmanager.com/CL0/http:%2F%2Fwww.plosone.org%2Fstatic%2Fpolicies.action%23sharing/1/010f0199f0164187-b2779843-ba5e-48b7-a0db-e390769d2d97-000000/1U8euLPKIzzKjv9DTSZ_qJUWLqyvvl4aHCbRFD_ki7A=233) requires authors to make all data underlying the findings described in their manuscript fully available without restriction, with rare exception (please refer to the Data Availability Statement in the manuscript PDF file). The data should be provided as part of the manuscript or its supporting information, or deposited to a public repository. For example, in addition to summary statistics, the data points behind means, medians and variance measures should be available. If there are restrictions on publicly sharing data—e.g. participant privacy or use of data from a third party—those must be specified.

Reviewer #1: Yes

Reviewer #2: Yes

4. Is the manuscript presented in an intelligible fashion and written in standard English?

PLOS ONE does not copyedit accepted manuscripts, so the language in submitted articles must be clear, correct, and unambiguous. Any typographical or grammatical errors should be corrected at revision, so please note any specific errors here.

Reviewer #1: Yes

Reviewer #2: Yes

5. Review Comments to the Author

Please use the space provided to explain your answers to the questions above. You may also include additional comments for the author, including concerns about dual publication, research ethics, or publication ethics. (Please upload your review as an attachment if it exceeds 20,000 characters)

Reviewer #1: This manuscript presents a novel Non-Fixed Paraffin Embedding (NFPE) method for pathological tissues that eliminates the use of both formalin and xylene, instead utilizing supercritical CO₂. The authors conduct a comprehensive evaluation of this technique across histochemistry, immunohistochemistry, and immunofluorescence, using a wide array of markers. The study is timely, relevant, and has significant implications for laboratory safety, environmental sustainability, and molecular diagnostics.

**Strengths:**
- The work clearly represents original research. While prior studies have explored either xylene-free or formalin-free approaches, this paper uniquely integrates both methods and validates the approach across a broad range of stains.
- The breadth of testing is impressive, encompassing 11 histochemical stains, 31 immunohistochemical markers, and 7 immunofluorescence markers.
- The results are generally strong: morphology is well-preserved, DNA quality is likely enhanced, and most markers functioned well with minimal protocol adjustments.
- The clinical and public health relevance is high, as the study provides a pathway to reduce exposure for pathology staff to toxic and carcinogenic substances.

**Concerns / Limitations:**
- **Marker Gaps:** Three immunohistochemical markers (CD68, PAX-8, TDT) did not produce reliable staining with NFPE. This limitation should be explicitly discussed in terms of diagnostic consequences and potential solutions, such as alternative antibody clones.

A: We now describe the relevance of these three markers and potential solutions in a new paragraph in the discussion of the revised manuscript.

- **Residual Formalin Use:** The reticulin staining required a brief dip in formalin. While this amount is minimal compared to traditional FFPE workflows, it undermines the claim of a completely formalin-free process. This nuance should be highlighted in the conclusions.

A: We now highlight this in the conclusions of the revised manuscript.

- **Statistical Analysis:** The evaluation is primarily descriptive and based on visual assessment. Incorporating quantitative scoring (e.g., inter-observer agreement, reproducibility measures, signal-to-noise quantification) would strengthen the robustness of the findings.

A: The assessments of all stainings were initially performed blinded by an expert reviewer (specialized technician), followed by a not-blinded assessment by a general pathologist (who assessed all stainings in this paper), and finally verified by a specialized pathologist. We now explain this in the methods section of the revised manuscript and show in tables 4, 5, and 6 that the inter-observer agreement is 100%. As mentioned above, we also quantified P63 signal intensity scoring between FFPE, NFPE, and XFPE groups and showed (in Figure S4) that we were able to achieve signal intensities that show no statistical difference.

- **Protocol Reproducibility:** While the authors note that protocols must be optimized for each laboratory, providing more detailed methodological information would assist other labs in reproducing and validating NFPE more effectively.

A: We have now extended the methods section in the revised manuscript and explained the NFPE process in detail.

- **Generalizability:** The study was conducted at a single institution. Broader validation across multiple pathology laboratories would be important before clinical adoption.

A: We agree 100% with the reviewer that broader validation across multiple pathology laboratories is very important to solidify our claims. We now mention this in the discussion of the revised manuscript. Several laboratories in the Netherlands already showed interest in the NFPE technique, and some bought Tispa machines to generate Xylene/Formalin free embedded tissues. We hope and expect that publication of this study will trigger much more interest in NFPE.

**Ethics, Integrity, and Reporting:**
- Ethical standards were met; residual anonymized tissue was utilized with confirmation from the ethics committee.
- The manuscript is well-written in clear English and is easy to follow.
- Data availability complies with journal standards, with supplementary data provided.

**Overall Recommendation:**
This is an important and highly original contribution that advances the field of pathology towards safer and more sustainable practices. With minor revisions—particularly clarifying limitations, expanding the discussion on marker gaps, and enhancing details on reproducibility—the manuscript will be a strong candidate for publication.

Reviewer #2: Niemantsverdriet et al. developed a novel method of tissue processing that eliminates the need for toxic chemicals xylene and formalin. They compared the efficacy of staining in non-formalin fixed tissues to the tissues processed with standard formalin-fixed methods. They tested variety of stains in different organs and they claim that they found comparable results in most of them. Overall, the manuscript is structured well. The methods need more detail, quantifiable parameters need to be used, and statistical analyses need to be performed to compare different groups. Because of lack of quantifiable measures, the conclusions drawn cannot be supported with data, despite histological images showing similar morphology. I have following questions and recommendations:
1. It is recommended that authors describe the NFPE processing method in this manuscript as well.

A: We have now extended the methods section and explained the NFPE process in detail.

2. Visual appearance of histological images stained with two different methods need not draw the same conclusions. I suggest adding a quantifiable metric to compare different methods. For example, do you see similar number of P63 positive cells in a tissue processed with FFPE and NFPE? Alternatively, you can develop a visual analog scale and have a blind reviewer assess the histological images of tissues with pathological conditions and confirm if similar inferences are made.

A: We now quantified P63 signal intensity scoring in FFPE, XFPE, and NFPE stainings as requested and show in a new supplementary figure (S4) that we were able to obtain P63 staining intensities in NFPE, XFPE, and FFPE tissues that show no statistical difference.

3. The authors should mention who drew the conclusions ‘Good morphology’. Were the conclusions drawn in a double-blinded manner by independent reviewers? Were they experts in identifying the morphology of the tissues? Were they pathologists?

A: The assessments of all stainings were initially performed blinded by an expert reviewer (specialized technician), followed by (not-blinded) assessment by a general pathologist (who assessed all stainings in this paper), and finally verified by a specialized pathologist. We now explain this in the methods section of the revised manuscript and show in tables 4, 5, and 6 that the inter-observer agreement is 100%. As mentioned above, we quantified P63 signal intensity scoring between FFPE, NFPE, and XFPE groups and showed that we were able to achieve signal intensities that show no statistical difference.

4. In NFPE H&E Skin images, collagen fibrils seem to have lost morphology. Is it consistent in all replicates? Will this technique (NFPE) work with picrosirius red staining with polarized light imaging? Will it be able to separate thin (green) and thicker (red) bundles?

A: In our laboratory and in the other diagnostic pathology laboratories in the Netherlands, the picrosirius red staining is not used for diagnostics. To assess collagen morphology in diagnostics, we use Azan, Masson Goldner (trichrome), and Elastica van Gieson (EvG) stainings. We performed these stainings on other tissue types in our original manuscript, but not yet on skin NFPE. In the revised manuscript, we performed these stainings on skin NFPE samples and added a new figure (Figure 2 in the revised manuscript). We describe the findings in a new paragraph. We did not observe altered (collagen) morphology.

5. I could not see information about the replicates. How many replicates were included per group?

A: The number of biological replicates is the number of different tissues used as specified in tables 4,5, and 6. For each tissue, we performed 2 technical replicates after optimization was completed. We now also explain this in the methods section of the revised manuscript.

6. There needs to be more discussion about XFPE. Clearly mention the significance and relevance of this group.

A: We added a paragraph to the discussion explaining the significance of XFPE for this study.

6. PLOS authors have the option to publish the peer review history of their article ([what does this mean?](https://track.editorialmanager.com/CL0/https:%2F%2Fjournals.plos.org%2Fplosone%2Fs%2Feditorial-and-peer-review-process%23loc-peer-review-history/1/010f0199f0164187-b2779843-ba5e-48b7-a0db-e390769d2d97-000000/qR-bxHcaHroFbjoYd6jwiHKhnOKa1pUOD5_KofuaCCY=233)). If published, this will include your full peer review and any attached files.

If you choose “no”, your identity will remain anonymous but your review may still be made public.

**Do you want your identity to be public for this peer review?** For information about this choice, including consent withdrawal, please see our [Privacy Policy](https://track.editorialmanager.com/CL0/https:%2F%2Fwww.plos.org%2Fprivacy-policy/1/010f0199f0164187-b2779843-ba5e-48b7-a0db-e390769d2d97-000000/JjFd9P8NtpLnJ8VydlSAyHaMWPZxtLtLgIv5S0pmRQg=233).

Reviewer #1: **Yes:** SADIK BAY

Reviewer #2: No

[NOTE: If reviewer comments were submitted as an attachment file, they will be attached to this email and accessible via the submission site. Please log into your account, locate the manuscript record, and check for the action link "View Attachments". If this link does not appear, there are no attachment files.]

While revising your submission, please upload your figure files to the Preflight Analysis and Conversion Engine (PACE) digital diagnostic tool, [https://pacev2.apexcovantage.com/](https://track.editorialmanager.com/CL0/https:%2F%2Fpacev2.apexcovantage.com%2F/1/010f0199f0164187-b2779843-ba5e-48b7-a0db-e390769d2d97-000000/rYJPW2ynol7pU9ndZiBLvbE5AltvRjV6w6RBm3BnDzY=233). PACE helps ensure that figures meet PLOS requirements. To use PACE, you must first register as a user. Registration is free. Then, login and navigate to the UPLOAD tab, where you will find detailed instructions on how to use the tool. If you encounter any issues or have any questions when using PACE, please email PLOS at [figures@plos.org](mailto:figures@plos.org). Please note that Supporting Information files do not need this step.
